# Probiotics in Experimental Ulcerative Colitis: Mast Cell Density and Neuronal Hypertrophy

**DOI:** 10.5152/tjg.2022.21550

**Published:** 2022-10-01

**Authors:** Arzu Hizay, Nigar Keleş-Çelik, Nuray Acar, Emine Mine Çomak-Göçer, Rahime Şekerci, Nuriye Öz, Ezgi Golal, Gülsüm Özlem Elpek

**Affiliations:** 1 Department of Anatomy, Akdeniz University Faculty of Medicine, Antalya, Turkey; 2 Department of Histology and Embryology, Akdeniz University Faculty of Medicine, Antalya, Turkey; 3 Department of Nutrition and Dietetics, Akdeniz University Faculty of Health Sciences, Antalya, Turkey; 4 Department of Pathology, Akdeniz University Faculty of Medicine, Antalya, Turkey

**Keywords:** Mast cell, probiotics, ulcerative colitis

## Abstract

**Background:**

Probiotics such as *Lactobacillus* and *Bifidobacterium* are among the supportive treatment methods to achieve effective results in ulcerative colitis. This study was established to investigate the effect of probiotics in experimental ulcerative colitis and to detect changes in mast cell and neuronal structures in this treatment method.

**Methods:**

A total of 48 adult male rats were used to study the effects of probiotics on ulcerative colitis. The animals were divided into 6 groups as control, experimental colitis, and four probiotic protective groups. Three different bacterial strains were administered to the protective groups individually and in combination by gavage. PGP 9.5 antibody and mast cell tryptase were used for the detection of neuronal structures and mast cells. The number of Schwann cells and ganglia, size measurements of ganglia, and density of mast cells were evaluated.

**Results:**

Compared to the control, an increase in the number of mast cells was detected in all groups. Especially the increase in the number of mast cells was found to be statistically significant in combined probiotic administration. In the detection of neuronal structures, a significant increase in the number of Schwann cells and ganglia was detected in groups where probiotics were administered combined and individually.

**Conclusion:**

These results suggest that probiotics may play a role in the supporting effect of increasing the number of mast cells and neuronal structures, protecting the intestinal wall. We think that more specific and detailed studies should be conducted to evaluate the protective/therapeutic effect of probiotics in future studies.

## Introduction

Ulcerative colitis (UC) is one of the types of inflammatory bowel disease (IBD) that occurs as a result of the interaction of various factors and causes inflammation in the gut. This condition results in inflammation of the intestinal mucosa; it causes ulceration, edema, bleeding, fluid, and electrolyte loss.^1^ There are many hypotheses for the etiopathogenesis of UC, but the mechanism is still unknown.

In recent years, studies have been conducted on the effects of mast cells and neuronal structures in the gastrointestinal system.^2^ Besides the inflammatory properties of mast cells, it also has effective roles such as restoration of tissues, vascularization, and cell proliferation. Various evidences about the effects of mast cells on Crohn’s disease and UC are available in the literature.^3,4^ However, the role of mast cell in experimental UC is still controversial. In colitis induced by trinitrobenzene sulfonic acid and dextran sodium sulfate, it was observed that the colitis was improved with mast cell deficiency,^5,6^ while in another study, it was found that the deficiency of these cells had no effect on colitis.^7^ Another study showed that mice deficient in mast cell protease-6 exhibited quenched colitis in experimental colitis models.^8^ It has been reported that mucosal damage in 2,4,6-trinitrobenzene sulfonic acid (TNBS) colitis is reduced by stabilization of mast cells and inhibition of proteases of these cells.^6^ In addition, although there is evidence to support the pro-inflammatory role of these cells in colitis, the beneficial effects of mast cells have also been reported in the literature. It has been reported that mast cells and protease II increase in rats with an experimental UC model. The researchers think that these cells may play a beneficial role in the healing process.^9^ In another study, delayed recovery was observed in mast cell-deficient mice in dextran sulfate sodium (DSS) colitis.^10^ Moreover, increased colitis in lack of IL-10/mast cell compared to lack of IL-10 suggests that these cells may have a protective role in colitis models in mice.^11^

When the intestinal lumen encounters a harmful agent, the mucosa is closely related and controlled by neuro-immune factors. With impaired control, gastrointestinal disorders and mucosal permeability occurs. Mast cells have been shown to play a role in the regulation of the mucosal barrier. Prevention of impairment of permeability in the mucosa via these cells is of interest at this time. Especially, it has been reported that probiotics and mast cell stabilizers show promising effects for patients with gastrointestinal disorders.^12^

Supplements of living microorganisms that are beneficial to the human intestine such as Lactobacillus, Bifidobacterium, Saccharomyces, and *Escherichia coli* are used as an auxiliary treatment method in intestinal disorders such as UC. Probiotics are thought to reduce pathogenic microorganisms and proinflammatory cytokines in the gut. However, it has been reported to improve its ability to act as a barrier in the gut.^13^

It has been reported that probiotics show significant advantages in intestinal permeability.^14^ In humans, probiotics have also been shown to have beneficial effects on remission in UC and maintenance therapy of pouchitis.^15^ On the contrary, there is a study reporting that the use of probiotics has negative effects and even increases mortality.^16^ Therefore, the mechanism of action of probiotics is quite complex and not clearly known. This study was created to investigate the effect of probiotic administration in rats with experimental UC and to determine the effect of this treatment on mast cell and neuronal structures.

## Materials and Methods

### Animals

In our study, 48 *Wistar *male rats with an average weight of 250-300 g were used. Animals were provided from the Akdeniz University Experimental Animals Unit. For the duration of the study, animals were supplemented with unlimited feed and water on a day and night cycle (12 hours’ light and 12 hours’ dark). All surgical procedures performed in our experiment were approved by the Akdeniz University Experimental Animal Ethics Committee (Ethics committee approval: 7793/October 28, 2018).

### Experimental Groups and Design

The animals were divided into 6 groups (n = 8 in each). Group 1 comprised control rats without colitis, used to determine the mast cells and neuronal structures in the colonic tissue. Group 2 consisted of only experimental colitis group. To evaluate the severity of colitis, colon tissue was taken 2 days after experimental colitis was established. In group 3, *Lactobacillus acidophilus *was given to each animal daily for 14 days in an amount of 10^9^ cfu (colony forming unit) in 1 mL distilled water by gavage. Ulcerative colitis model was applied to these animals after light ether anesthesia on the 15th day and the intestinal tissues were taken by sacrificing the subjects on the 17th day. In group 4, *Bifidobacterium bifidum *was given to each animal daily for 14 days in an amount of 10^9^ cfu in 1 mL distilled water by gavage. Ulcerative colitis model was applied to these animals after light ether anesthesia on the 15th day and the intestinal tissues were taken by sacrificing the animals on the 17th day. In group 5, *Lactobacillus rhamnosus *was given to each animal daily for 14 days in an amount of 10^9^ cfu in 1 mL distilled water by gavage. Ulcerative colitis model was applied to these animals after light ether anesthesia on the 15th day and the intestinal tissues were taken by sacrificing the animals on the 17th day. In Group 6, Cocktail probiotics (*L. acidophilus* + *B. bifidum* + *L. rhamnosus*) was given daily for 14 days to each animal at an amount of 3 × 10^9^ cfu in 3 mL distilled water by gavage. Ulcerative colitis model was applied to these animals after light ether anesthesia on the 15th day and the intestinal tissues were taken by sacrificing the animals on the 17th day (Table 1).

### Probiotics Strain

The reference strains, *B. bifidum *(DSM 20456), *L. acidophilus *(DSM 20079) were supplied from German Collection of Microorganisms and Cell Cultures GmbH, Braunschweig, Germany. *L. rhamnosus *GG was provided by Chr. Hansen A/S (Horsholm, Denmark).

Probiotic strains were inoculated into MRS broth (DE MAN, ROGOSA and SHARPE for microbiology) to an initial optical density at 600 nm (OD600) of 0.1 and anaerobically incubated for 24 hours at 37°C. After anaerobic incubation, cell pellet was collected by centrifugation (13 000 g, 5°C, 5 minutes), washed 2 times in sterile water, and re-suspended in sterile water for adjusted to a cell density of 10^9^ cfu/mL.

### Acetic Acid Colitis Model

Experimental UC was performed intrarectally with acetic acid (4%, 2 mL, pH 2.4). Under the mild ether anesthesia, a soft pediatric catheter was placed rectally reaching 6-7 cm forward. The animals were placed in the Trendelenburg position during this procedure. After the administration, rats were kept in the Trendelenburg position for 30 seconds.^17^

### Histologic Assessment

Hematoxylin and eosin H&E staining was used as a histological evaluation method to evaluate inflammation in the colon mucosa. Colonic samples were fixed with formalin, they were embedded in paraffin blocks, and 7 μm sections were taken. Tissue sections were evaluated by the pathologist with the inflammatory activity score. This scoring was from 0 to 3 (0; normal, 1; mild, 2; moderate, 3; severe). In inflammation, the number of inflammatory cells in the lamina propria, the thickness of the muscle layer, and the damage to the mucosa were evaluated.

### Immonohistochemical Staining

Tissues were fixed in formalin and embedded in paraffin. Tissues were cut into 5 µm sections and placed on poly-l-lysine-coated slides (#J1800AMNZ, Thermo Scientific, Braunschweig, Germany). After the incubation, sections were rehydrated through a decreasing gradient of ethanol, rinsed in distilled water, and washed 3 times in the phosphate-buffered saline (PBS, pH 7.2-7.4). Antigen retrieval procedure was performed by treating the sections in 10 mM citrate buffer (pH 6.0) (#100242, Merck, Darmstadt, Germany), in a microwave oven at 600 W for 5 minutes, 3 times. After cooling for 20 minutes at room temperature, the sections were washed in PBS (pH 7.4) for 5 minutes. Endogenous peroxidase activity was blocked by incubation in methanol containing 3% H_2_O_2_ (#108597, Merck) for 15 minutes and washed with PBS 3 times. Afterward, sections were incubated with a blocking agent (UV Block, #TA-125UB, LabVision Corporation, Fremont, Calif, USA) for 7 minutes at room temperature. Excess serum was removed, and sections were incubated with rabbit polyclonal anti-PGP9.5 (#ab10404; Abcam) at 1:500 dilution and mouse monoclonal anti-mast cell tryptase (#ab2378, Abcam) at 1:750 dilutions at +4°C overnight. The primary antibodies were substituted with normal rabbit IgG (#sc-2027, Santa Cruz Biotechnology, Santa Cruz, Calif, USA) and normal mouse IgG (#sc-2027, Santa Cruz Biotechnology, Santa Cruz, Calif, USA) in the same dilutions as the specific antibodies. Next day, sections were washed with PBS for 3 times. Then, they were incubated with biotinylated goat anti-rabbit (#BA-1000, Vector Laboratories, Burlingame, Calif, USA) and biotinylated goat anti-mouse antibody (#BA-9200, Vector Laboratories) at 1:500 dilutions in a humid environment at room temperature for 45 minutes, followed by incubation with horseradish peroxidase-conjugated streptavidin complex (#TS125HR, Thermo Scientific, Fremont, Calif, USA) for 20 minutes at room temperature. Sections were washed 3 times with PBS. Sections were treated with Di Amino Benzidine chromogen (#D4168, Sigma Aldrich, St. Louis, MO, USA) for 5 minutes to improve signaling and then washed in tap water. Mayer’s Hematoxylin (#109249 Merck, Darmstadt, Germany) was utilized for counterstaining the sections, and they were dehydrated and mounted with Entellan (#107961; Merck, Darmstadt, German) mounting medium.

### Digital Image Analysis

SAMBA image analysis (Alcatel-TITN, Grenoble, France) was used in our study. This image analysis includes a microscope (Leitz Diaplan Microscope) connected to a personal computer. The slides were examined at 100× magnification. Neuronal structures and mast cells were counted and photographed at 200× magnification.

### Statistical Analysis

In the analysis of data, descriptive statistics are presented with mean and standard deviation values. Kruskal–Wallis test was used to analyze the difference between Schwann cell number, Gang (N) cell number, Gang (A) cell number, and mast cell number measurement values of the groups. Mann–Whitney *U* comparison test was used for each paired group to determine the groups that made the difference. *P* values less than .05 were considered statistically significant in the study. Analyses were made with Statistical Package for the Social Sciences 22.0 package program (IBM Corp.; Armonk, NY, USA).

## Results

### Histologic Assessment

**Figure 1.**
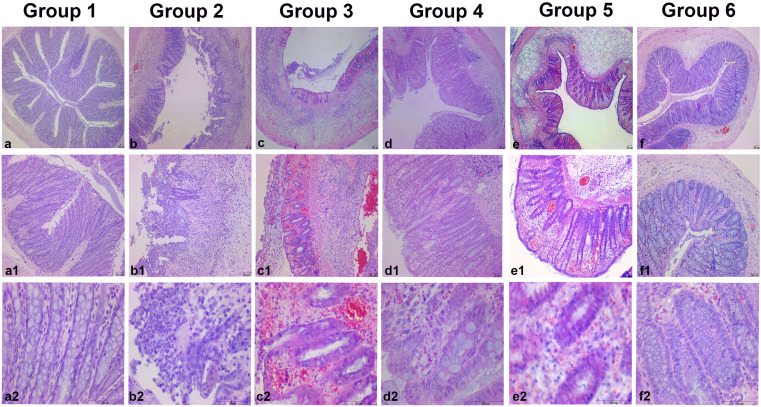
Hematoxylin and eosin staining of colon descendens of experimental groups’. Scale bars: 50 µm. Figures a-f 40×, a1-f1 100×, a2-f2 400× magnification.

To determine the type of inflammation in colon tissues, H&E staining was performed. Mucosal destruction, the number of inflammatory cells in the lamina propria, and muscular layer thickness were evaluated with the inflammatory activity score in the colon tissue sections. Compared to the control group (group 1), a significant and severe inflammation was detected in the UC group (group 2), including inflammatory cell infiltration, muscle layer thickness, and mucosal destruction (Figure 1). In group 2, the deterioration of the villi structure and thickening of the muscle layer were significantly observed compared to the control (*P* < .05) (Figure 1/b, b2). In colitis after probiotic treatment (groups 3-6), the mucosal layer was more regular and the muscular layer thickness was closer to the control group. Inflammatory cells were observed in the colon sections of these groups, and a significant decrease was detected compared to group 2 (*P* < .05). No statistically significant difference was found between the individual probiotic treatment (group 3, 4, and 5) (*P* > 05). Muscle layer thickness and mucosal damage were significantly reduced in group 6, where probiotics were given in combination, compared to the other groups (*P* < .05). The structure of the villi was regular in tissues in this group compared to groups 2-5 (Figure 1). As a result, macroscopic examination of the colon and estimates of its thickness clearly showed that the inflammation caused by acetic acid was more pronounced in the UC group (group 2) compared to the control. It revealed that the colonic mucosa exposed to colitis was markedly infiltrated with polymorphonuclear leukocytes. This cellular infiltration caused the thickness of the wall of the colon to increase, particularly in group 2. The inflammatory reaction was also seen in the colon tissue of all groups that received probiotics, but was less severe.

### Mast Cell Density

**Figure 2.**
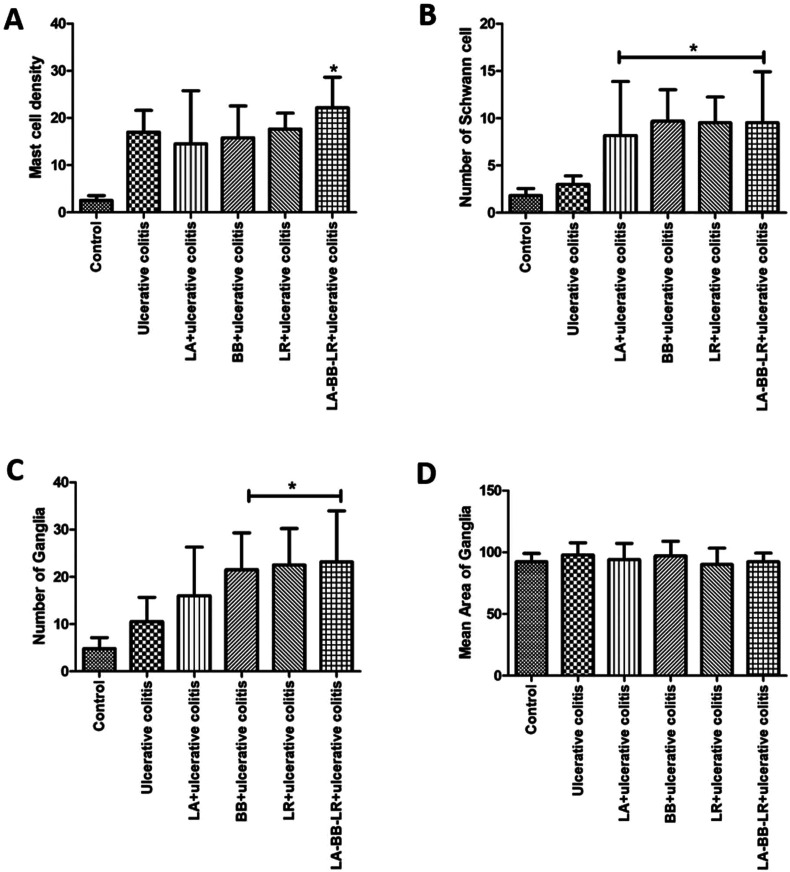
Diagram showing mast cell density, Schwann cell number, number of ganglia, and mean area of ganglia. (A) Mast cell density. *Indicates a significant difference compared to the control and ulcerative colitis group (group 2) (*P* < .05). (B) Number of Schwann cell. *Indicates a significant difference compared to the control and ulcerative colitis group (group 2) (*P* < .05). (C) Number of ganglia. *Indicates a significant difference compared to the control and ulcerative colitis group (group 2) (*P* < .05). (D) Mean area of ganglia. There was no statistically significant difference between the groups and compared to the control group.

Mast cell tryptase was used for the detection of mast cells by immunohistochemical method. It has been determined that mast cells are triggered in the intestinal mucosa and there is an increase in experimental ulcerative groups (Figure 2).

**Figure 3.**
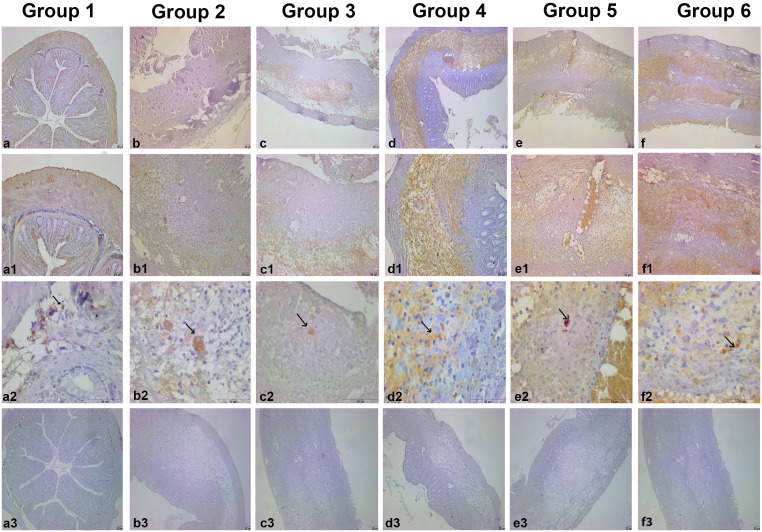
Mast cell tyrptase immunohistochemistry in colon descendens of experimental groups. Scale bars: 50 µm. (a-f) 40×, (a1-f1) 100×, (a2-f2) 400× magnification. (a3-f3) 40× magnification and show isotype controls. Black arrows show mast cells.

Compared to the control group, an increase in the number of mast cells was detected in all groups. Statistically significant differences were detected in the cocktail probiotic + UC (group 6) group compared to groups 1 and 2 (*P* < .05). There is also an increase in mast cell density in groups where probiotics are administered individually (groups 3, 4, and 5) compared to the control group. However, this increase was not detected as a significant difference between these groups (Figures 1 and 3) (*P* > .05).

### PGP 9.5 Immunoreactivity/Schwann Cell Density

It was observed that the PGP 9.5 positive Schwann cells were distributed throughout the submucosa and muscular layer at different levels (Figure 2). Schwann cell number was significantly higher in groups 3-6 compared to the control (group 1) and experimental ulcerative group (group 2) (*P* < .01). The number of Schwann cells in group 1 and group 2 was found to be 1.83 ± 0.75 and 2.10 ± 0.50, respectively. In groups 3 to 6 were determined as 8.17 ± 5.71, 9.67 ± 3.33, 9.50 ± 2.74, and 9.50 ± 5.43, respectively (Figures 2 and 3). When the results were evaluated, in colitis after probiotic treatment (given individually and in combination), significantly increase in Schwann cells was observed.

### PGP 9.5 Immunoreactivity/Number and Area of Ganglia (N)

**Figure 4.**
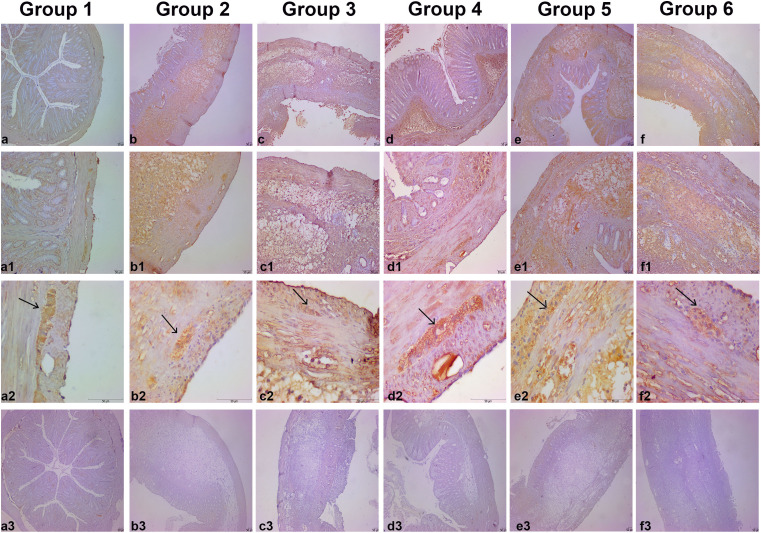
PGP 9.5 immunohistochemistry in colon descendens of experimental groups. Scale bars: 50 µm. (a-f) 40×, (a1-f1) 100×, (a2-f2) 400× magnification. (a3-f3) 40× magnification and show isotype controls. Black arrows show Schwann cells.

It was determined that the number of Gang (N) cells was different in all groups. These neuronal structures increased in all groups compared to the control (Figure 2). However, a significant difference was detected in group 4 (21.50 ± 7.82), group 5 (22.50 ± 7,71), and group 6 (23.17 ± 10.80) compared to the control (4.83 ±2.32) and UC group (10.50 ± 5.17) (*P* = .001). In group 3 (*L. acidophilus* (LA) + experimental UC), there was no significant difference compared to the control (*P* > .05) (Figures 2 and 4).

**Figure 5.**
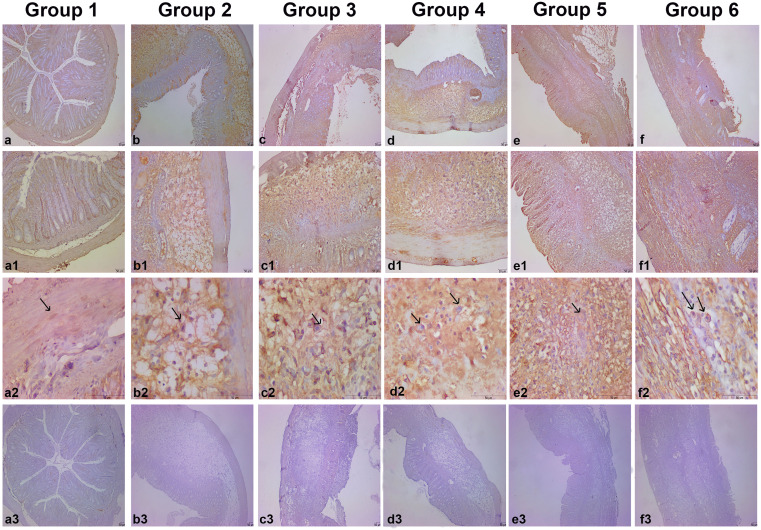
PGP 9.5 immunohistochemistry in colon descendens of experimental groups. Scale bars: 50 µm. (a-f) 40×, (a1-f1) 100×, (a2-f2) 400× magnification. (a3-f3) 40× magnification and show isotype controls. Black arrows show ganglion cells.

It was observed that the number of Gang (A) was not at different levels according to the groups. It was found that the number of these cells in the intestinal mucosa was at similar levels in all experimental groups compared to the control (*P* = .51) (Figures 2 and 5).

## Discussion

In this study, we investigated the effect of probiotics on mast cells and neuronal structures in acetic acid-induced colitis inflammation. It was determined that mast cells were triggered and there was increase in experimental UC groups. Compared to the control and UC group, the increase in the number of mast cells was found to be statistically significant in combined probiotic administration. In the detection of neuronal structures, the increase in Schwann cells and number of ganglia show a significant increase in the groups where probiotics are administered in combination and individually. The significant increase in mast cell and neuronal structures in receiving ulcerative colitis model following probiotic treatment suggests that probiotics may have a protective role in the intestinal wall.

The best-known role of mast cells is in fighting allergic diseases, but recent studies have emphasized the role of these cells in protecting against infection.^18^ These cells, which play a protective role against pathogens, secrete various mediators in cases such as IBD. They also play a role in wound repair and tissue remodeling.^19^ These irreversible and destructive processes are thought to be associated with delayed wound healing. Mast cells and their mediators are important factors in wound healing processes. It has been reported that tryptase-expressing mast cells provide colon healing in colitis, and this tryptase expression continues until the 20th day after colitis.^19^

It is known that mast cells accumulate in intestinal lesions in conditions such as IBD, ulcerative colitis, celiac disease, and Crohn’s disease.^20^ These cells interact with the nervous system as well as with microbiota by sending signals to enteric neurons via serotonin. However, it responds rapidly to the abnormality of the intestinal flora and its permeability to the epithelium by releasing some mediators in the inflammatory reaction.^21^

Although it is known as the modulator of immunosuppression and inflammation, the effects of mast cells in gastrointestinal diseases are not fully known. The density of mast cell increases in the gastrointestinal tract of IBD patients. This indicates that mast cells play a possible role in the etiology of diseases.^2^ In one study, lack of mast cell has been reported to increase inflammation in experimental colitis because it affects body weight and hypersensitivity.^8^ In addition, mast cells have been shown to mediate epithelial barrier dysfunction in stress-induced intestinal inflammation and decreased intestinal permeability in lack of mast cell in mice.^22^ Lack of IL-10 and mast cell has been reported to cause an increase in mucosal epithelial permeability but does not have an inhibitory role in the intestinal inflammatory response in mice.^23^ Although it has been reported in the literature that mast cells have a pro-inflammatory effect in colitis, there are more recent studies showing that severe inflammation occurs in the lack of these cells.^24^ In our study, histologic observation showed that invasion of the inflammatory cells in the mucosa was prominent in the colonic sample. In addition, in the inflamed area, number of mast cells in the colon mucosa was increased in the UC model induced with acetic acid.

It is thought that anomalies or exacerbation of diseases occurring in the gastrointestinal system are also related to microbiota regularity. It is available in the literature that it interacts with the microbiota by activating mast cells. The effects of bacteria on the activation and function of these cells have been extensively reported in the literature, with conflicting results.^24^

Studies showing that treatment with probiotics provides advantages in intestinal mucosal permeability have been conducted in animal models.^25^ In humans, probiotics have also shown positive effects on maintenance therapy and remission of pouchitis in UC.^15^

In our study, we evaluated the effects of 3 different probiotics on mast cell and neuronal structures in groups with UC. We administered probiotics individually and in combination by oral gavage before colitis. When the data obtained were evaluated, we observed an increase in mast cells, Schwann cells, and ganglia in all groups that we applied probiotic treatment. We found a statistically significant increase especially in the group where probiotics were applied in combination. With this result, we think that probiotic therapy may have a protective role in the intestinal tissue, and these cells may have multiple roles under different conditions with the increase in the number of mast cells. These cells can act as anti-inflammatory mediators in the intact immune system or suggest they may have protective roles under risky conditions.

There is evidence that mast cells interact with peripheral nerves in many tissues.^26^ In the gastrointestinal tract, mast cells are closely linked to nerves and are thought to be necessary for nerve growth and repair.^27^ It has also been shown that the enteric nervous system (ENS) and mast cells play a role during bowel inflammation.^28^ Although mast cells play a role in the local regulation of immune events, there is increasing evidence that these cells can affect nerve function and remodeling during inflammation with their number, distribution, and content of chemical mediators. In the gastrointestinal tract, bidirectional communication between mast cells and ENS has been described.^29^ An association of mast cells and hypertrophic nerve fibers has been identified in chronic IBDs such as Crohn’s disease and UC.^30^

The effect of probiotics on the neuronal hypertrophy and mast cell in UC has not been reported yet. In the present study, it has been demonstrated that mast cell increased with the administration of probiotics in UC and highly correlated with an increased PGP 9.5 positivity, the number of Schwann cells, and ganglia.

In conclusion, treatment with probiotics may protect against intestinal dysfunction and mucosal infiltration by altering immune mediators and mast cells. The mechanisms underlying mediator release in response to probiotics are unknown, and the mechanisms that affect the response of probiotics to mast cell and neuronal structures should be explained in future studies. There is clear evidence of the role of mast cells in regulating the immune and mucosal integrity of the intestines, and mast cells could possibly be considered as one of the potential mediators of the mechanism of action of probiotics. In summary, mast cells are involved in high numbers or secreted mediators in many inflammatory conditions such as allergies, atherosclerosis, and IBDs. Most studies have found that mast cells exhibit their pro-inflammatory effects, but suppressive, regulatory, and anti-inflammatory mast cell reactions have also occurred. With future studies, the protective roles of mast cells can be fully elucidated, and regulation of mast cell activation may become increasingly important in the management of inflammatory diseases.

## Figures and Tables

**Table 1.** Experimental Groups

Groups	n
**Group I.** Control	8
**Group II.** Experimental ulcerative colitis	8
**Group III**. *Lactobacillus acidophilus* (LA) + experimental ulcerative colitis	8
**Group IV.** *Bifidobacterium bifidum* (BB) + experimental ulcerative colitis	8
**Group V.** *Lactobacillus rhamnosus* (LR) + experimental ulcerative colitis	8
**Group VI.** Cocktail probiotics (LA +BB + LR) + experimental ulcerative colitis	8
